# Effect of polymorphisms in the *CD36* and *STAT3* genes on different dietary interventions among patients with coronary artery disease: study protocol for a randomized controlled trial

**DOI:** 10.1186/s13063-016-1564-1

**Published:** 2016-09-05

**Authors:** Vera Lucia Portal, Melissa Medeiros Markoski, Alexandre Schaan de Quadros, Sílvia Garofallo, Julia Lorenzon dos Santos, Aline Oliveira, Camila Wechenfelder, Viviane Paiva de Campos, Priscilla Azambuja Lopes de Souza, Luana Machado, Aline Marcadenti

**Affiliations:** 1 Postgraduate Program in Health Sciences: Cardiology, Institute of Cardiology of the Rio Grande do Sul (IC/FUC), Porto Alegre, Brazil; 2 Department of Nutrition, Federal University of Health Sciences of Porto Alegre (UFCSPA), 245 Sarmento Leite Street, Porto Alegre, RS 90050-170 Brazil

**Keywords:** Coronary artery disease, Biological markers, Polymorphism, Genetic, Nuts, Olive oil, Randomized clinical trial

## Abstract

**Background:**

Cardiovascular disease has become a major health problem, and it has been associated with both environmental and genetic factors. Studies have shown that the Mediterranean Diet (MeDiet), or its components such as nuts and olive oil, may be strongly associated with the improvement of cardiovascular risk factors in specific populations. The purpose of the GENUTRI study is to investigate the interaction of genetics with cardiovascular risk factors in a non-Mediterranean population with coronary artery disease (CAD) according to three different diets: rich in pecan nuts, in extra-virgin olive oil or a control diet.

**Methods/design:**

The GENUTRI study is a single-center, randomized, open-label, parallel-group, 12-week pragmatic clinical trial conducted in patients aged 40 to 80 years and diagnosed with CAD. A standardized questionnaire will be applied to data collection and a blood sample will be obtained for lipid, glycemic and inflammatory profile evaluation. Polymorphisms in the *CD36* and *STAT3* genes will be detected using the TaqMan® SNP Genotyping Assay. Patients will be allocated in three groups: group 1: 30 g/day of pecan nuts; group 2: 30 ml/day of olive oil; and group 3: control diet. The primary outcome will consist of changes in LDL-cholesterol (in mg/dl) after 12 weeks of intervention.

**Discussion:**

Studies have shown the beneficial effects of diets rich in nuts and olive oil mainly in the Mediterranean population. GENUTRI is a clinical trial focusing on the effects of nuts or olive oil supplementation in Brazilian individuals. Additionally, we will try to demonstrate that genetic polymorphisms linked to cardiovascular disease may modulate the effects of different diets on biochemical and inflammatory markers among these subjects.

**Trial registration:**

ClinicalTrials.gov Identifier: NCT02202265 (registered on 18 July 2014: first version).

## Background

According to the World Health Organization (WHO), cardiovascular disease is the leading cause of mortality worldwide, being responsible for 18 million deaths annually [1]. Coronary artery disease (CAD) is considered the main cardiovascular disorder, and approximately 46 % of the cardiovascular mortality in men and 38 % in women is attributed to CAD [2].

About 80 % of all mortality from cardiovascular disorders could be prevented if obesity, unhealthy diet and physical inactivity were excluded from the general population [3] and the adherence to a higher-quality diet is associated with decreased mortality risk [4]. In this meaning, the Mediterranean Diet (MeDiet), which incorporates the traditional healthy living habits of people from countries bordering the Mediterranean Sea, has been consolidated as a dietary pattern highly associated with reduced cardiovascular [5] and mortality risk [6]. Besides, adherence to a MeDiet pattern seems to be strongly related to cardiovascular protection when compared to low-fat dietary patterns [5, 7].

Nuts (rich in polyunsaturated fatty acids – PUFA) and olive oil (rich in monounsaturated fatty acids – MUFA) are highly used in the Mediterranean cuisine [8]. Despite regular intake of nuts and olive oil seems to be inversely associated with cardiovascular risk factors, CAD and mortality in some populations [9–11], these effects have not been observed in all studies, mainly in non-Mediterranean countries [12, 13]. The PREDIMED (PREvención con DIeta MEDiterránea) study was a larger clinical trial that randomly assigned participants who were at high cardiovascular risk (but with no cardiovascular disease) to one of three diets: a MeDiet supplemented with extra-virgin olive oil (minimum 50 ml/day) or with mixed nuts (30 g/day), or a control diet (low-fat diet). The PREDIMED study showed that both MeDiets supplemented with extra-virgin olive oil or nuts reduced, by approximately 30 %, the incidence of major cardiovascular events in a European population [5].

Beyond unhealthy diet, genetic factors are strongly associated with the development and the progression of cardiovascular diseases [14]. The *CD36* (CD36 molecule (thrombospondin receptor)) and the *STAT3* (signal transducer and activator of transcription 3 (acute-phase response factor)) genes are both related to pathways involved in the genesis of CAD [15, 16], and may interact with dietary factors. The single-nucleotide polymorphism (SNP) rs1761667 G>A in the *CD36* gene has been associated with a higher intake of dietary fats and obesity [17], and the SNP rs8069645 A>G in the *STAT3* gene seems to interact with higher saturated fat intake and increases the risk for abdominal obesity [18]. However, the association of these SNPs with cardiovascular risk factors, such as lipid profile and inflammatory markers, and a possible interaction with different dietary fats are poorly understood in patients with CAD.

Therefore, we design a randomized clinical trial to investigate the interaction of genetics with cardiovascular risk factors in subjects with CAD who do not live in the Mediterranean region, according to dietary prescription with different supplementary fats used in the MeDiet.

## Methods/design

### Study design and centers

The GENUTRI study is a single-center, randomized, open-label, parallel-group, 12-week pragmatic clinical trial (blinded outcome assessment). The study population will include patients with the diagnosis of CAD identified during a period of hospitalization in the Institute of Cardiology of Rio Grande do Sul (IC/FUC), Brazil, and outpatients who volunteered for the trial. The randomization, allocation and follow-up procedures will take place at the outpatient Clinic of Nutrition.

### Study objectives

The primary objective of this randomized-controlled clinical trial is to evaluate the effect of three dietary approaches on metabolic, inflammatory and anthropometric profiles in patients with CAD after 12 weeks. The secondary objective is to detect an interaction of patients’ response to these three dietary approaches with polymorphisms in the *CD36* and *STAT3* genes.

### Participants

Inclusion criteria: patients aged 40–80 years diagnosed with CAD who have signed an informed consent to participate. CAD was defined by a diagnosis of previous myocardial infarction, acute coronary syndrome or typical angina pectoris. Myocardial infarction was defined according to the universal definition of myocardial infarction [19].

Exclusion criteria: psychiatric disease; morbid obesity (body mass index (BMI) ≥40 mg/m^2^); expectancy of life less than 6 months; pregnancy or lactation; renal failure (in dialysis); congestive heart failure; prior organ transplantation; uncontrolled hypo/hyperthyroidism; wheelchair-dependant individuals; use of vitamin/nutritional supplements; chronic use of nonsteroidal anti-inflammatory drugs; and concomitant participation in another experimental study.

### Ethical aspects

All procedures will be conducted in accordance with the ethical standards for human subject research set forth in the Declaration of Helsinki and also with the Good Clinical Practice (GCP) guidelines [20]. Written informed consent will be obtained by researchers from all patients included in the trial. The project was approved by the Research Ethics Committee of the Institute of Cardiology (registration number UP. 4861.13) and is registered in the ClinicalTrials.gov database.

### Study protocol

Patients with CAD who have been hospitalized in our institution and/or those submitted to coronary interventions for CAD will be invited by telephone calls to participate in the study. For the individuals who meet the inclusion criteria and are eligible for the trial, an appointment will be scheduled with the researchers’ staff (physicians and nutritionists). Voluntary individuals who contact the investigators and who meet the inclusion criteria will be also scheduled. All individuals will be advised by telephone to fast overnight for 8 hours before the meeting.

After checking all exclusion criteria and signing the informed consent, a blood sample will be collected and the subjects will undergo a baseline cardiology assessment. A structured questionnaire containing demographic, socioeconomic and educational data will be administered. Medical history (including previous outcomes and drug prescriptions) and behavior data, such as smoking, alcohol intake and level of physical activity will be collected. Blood pressure levels will be evaluated; anthropometric data (weigh, height, waist, hip and neck circumferences) and records about actual dietetic consumption will be assessed.

**Fig. 1:**
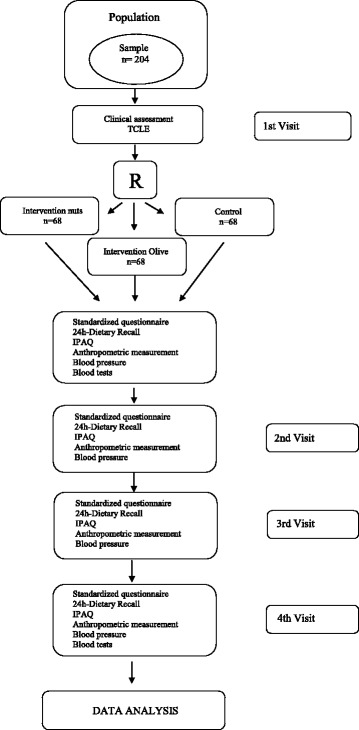
Flow chart of the GENUTRI study

After randomization and once allocation has been completed, the patient will be appropriately advised according to the intervention designated: pecan nut supplementation diet, olive oil supplementation diet or a control diet. Patients will be followed for a period of 3 months (12 weeks), and follow-up visits will be scheduled at 30 days, 60 days and 90 days (final appointment), when blood will be collected for laboratory analyses. Figure 1 shows all procedures and its periodicity during the trial according to follow-up visits. The patients will be reminded and prompted regarding the follow-up visits and the interventions by telephone calls.

### Randomization and blinding

The randomization sequence (in blocks) will be computer-generated using the http://www.randomization.com/ website, and then organized in sealed opaque envelopes. After fulfilling the eligibility criteria during the first visit, participants will be randomly assigned by independent investigators to one of the three diet groups in accordance with the generated randomization list. Both patients and investigators responsible for evaluation and data collection will be aware of the assigned diet. The staff involved in the biochemical outcomes’ evaluation and in the statistical analysis will be blinded to group allocation.

### Interventions

All individuals will receive individual dietary advice according to their specific caloric needs (to maintain or to lose weight) despite treatment group, and the following recommendations will be used to calculate the total daily calories: to weight management: 25 kcal/kg/day; to weight loss: 20 kcal/kg/day [21]. The individual distribution of macronutrients (dietary carbohydrates, protein and fats), independent of the group allocated, will be determined and calculated according to Brazilian guidelines for dyslipidemia [22], metabolic syndrome [21], hypertension [23] and diabetes [24].

Group 1 (nut supplementation, NS) will receive, in addition to dietary prescription, 1 kg of pecan nuts adequately packed for 30 days of treatment (approximately 900 g, considering an intake of 30 g/day [25]) plus educational materials concerning the adequate ingestion and storage of the nuts. Patients will be counseled about not consuming olive oil during the treatment. Group 2 (olive oil supplementation, OS) will receive, in addition to dietary prescription, 1 liter of extra-virgin olive oil adequately packed for 30 days of treatment (approximately 900 ml, considering an intake of 30 ml/day [25]) plus educational materials concerning the adequate ingestion and maintenance of the olive oil. Patients will be advised to not consume mixed nuts and oilseeds during the study period. Group 3 (control diet, CD) will receive standard dietary advice according to Brazilian guidelines [21–24] and will be discouraged from using olive oil, nuts and oilseeds during this period.

### Assessment tools and variables

#### Demographic variables

A structured questionnaire will be administered to all participants for collection of demographic parameters (age, sex, ethnicity), socioeconomic and educational data.

#### Genetic data

Deoxyribonucleic acid (DNA) will be isolated from a 5-ml venous blood sample by means of a specific toolkit according to the manufacturer’s instructions. The detection of polymorphisms and genotype characterization will be performed from frozen blood samples at −80 °C by means of the TaqMan® SNP Genotyping Assay according to the manufacturer’s instructions and to the available protocol. For each gene, a different genotyping assay kit will be used: ID C_30301828_10 to detect the G allele in rs8069645 *STAT3* and ID C_ 8314999_10 to detect the A allele in rs1761667 *CD36*.

#### Clinical and behavioral variables

Medical history: data on CAD specifications, history of present illness, past medical history, history of previous outcomes (i.e., heart attack and stroke) and current medications will be collected during the patient interview. Hypertension, type-2 diabetes mellitus and dyslipidemia will be considered present according to previous diagnosis and/or the use of medications to treat each of these conditions.

Blood pressure: systolic and diastolic blood pressure will be evaluated using an automatic validated monitoring device (OMRON® HEM-7200) with an adequately sized cuff to the arm circumference, according to the guidelines [23].

Body weight and height: weight (kg) will be measured with participants barefoot, wearing minimal clothing, and standing at the center of a digital platform scale; height (cm) will be obtained with participants barefoot in the standing position, and with both arms hanging freely at the side with palms facing thighs.

Waist, hip and neck circumferences: waist circumference (cm) will be obtained with a plastic, flexible measuring tape at the middle point between the lower costal margin and the iliac crest in a perpendicular plane, with the patient standing on both feet, approximately 20 cm apart, and with both arms hanging freely. Hip circumference will be measured at the level of the widest circumference over the buttocks with the research assistant kneeling at the side of the participant, so that the level of maximum extension can be seen. Neck circumference (cm) will be measured with the head straight and eyes staring forward, horizontally, 1 inch above the laryngeal prominence.

Smoking and alcohol intake: smoking will be defined as a categorical variable (never, current or past smoking) and excessive alcohol intake will be detected if participants have a consumption of 30 g or more and 15 g or more of ethanol a day for men and women, respectively [26].

Dietary evaluation: food intake will be evaluated by means of 24-h dietary recalls (24-HDRs). Caloric intake and nutrients will be estimated by Avanutri Revolution® Nutritional Evaluation software (Rio de Janeiro, Brazil) [27].

Physical activity: levels of physical activity will be evaluated according to the International Physical Activity Questionnaire (IPAQ) short version [28] translated and validated into the Portuguese language.

#### Laboratory variables

Blood samples will be collected by a trained professional at baseline and after 12 weeks. Samples will be centrifuged at 22 °C, 2000 rpm, for 10 min and stored in Eppendorf tubes at −80 °C for later analysis.

Lipid profile: total cholesterol, low-density lipoprotein cholesterol (LDL-c) and serum triglycerides will be evaluated by the enzymatic colorimetric method; high-density lipoprotein cholesterol (HDL-c) will be evaluated by the immunoprecipitation method (Roche Modular P Chemistry Analyzer®).

Inflammatory profile: interleukin-6 (IL-6), interleukin-10 (IL-10) and tumor necrosis factor alpha (TNF-α) will be evaluated by enzyme-linked immunosorbent assay (ELISA) using commercial kits (R&D Systems, Inc., Minneapolis, MN, USA), and according to the manufacturer’s instructions in a spectrophotometer (SpectraMax M2®). High-sensitivity C-reactive protein (hs-CRP) will be evaluated by the turbidimetric technique (Roche Cobas Integra 400 Plus Chemistry Analyzer®). Fibrinogen will be evaluated by the coagulometric method (Sysmex CA-600 systems®).

Glycemic profile: fasting glucose will be evaluated by the enzymatic colorimetric method (Roche Modular P Chemistry Analyzer®). Glycated hemoglobin will be evaluated by the immunoturbidimetric method (Roche Cobas Integra 400 Plus Chemistry Analyzer®). Serum insulin will be evaluated by electrochemiluminescence technique (Roche Elecsys 2010 Immunoassay Analyzer®).

### Outcome measures

#### Primary outcome

The primary outcome measure will consist of changes in LDL-c (mg/dl) from baseline to 12 weeks.

#### Secondary outcomes

- Changes in other lipid measures (total cholesterol, HDL-c, serum triglycerides) and inflammatory profiles (hs-CRP, IL-6, IL-10 and TNF-α) from baseline to 12 weeks.
- Changes in glycemic profile (fasting glucose, serum insulin and glycated hemoglobin), anthropometric indexes (weight, BMI and waist and neck circumferences) and in systolic and diastolic blood pressure from baseline to 12 weeks.

Patients will be prompted to complete the follow-up; if this is not possible, a 12-week visit will be scheduled for blood collection and measurements of primary and secondary outcome variables.

### Sample size

Sample size calculation was carried out in the WinPepi 11.20 for Windows program (Brixton Health, Israel). We used the data reported by Casas et al. [9], which assessed the effectiveness of a Mediterranean Diet pattern compared to a control diet among 164 individuals at high risk for cardiovascular disease. For a significance level of *α* = 0.05 and a statistical power of 80 %, with a between-group difference corresponding to a reduction of 13 mg/dl in the primary outcome (LDL-c) and considering no differences between olive oil and nut diets, the minimum sample size would be 171 patients (57 randomized to each group). Considering an expected loss rate of 20 % of patients, the total sample size needed will be 204 patients.

The allele frequency of the rs1761667 G>A and rs8069645 A>G SNPs in a Brazilian population is unknown. Since, in other studies, a frequency of approximately 40 % for each allele (A and G) has been found [23, 24], we expect that 204 individuals will provide a statistical power of 95 % in this analysis.

### Statistical analyses

All data will be double-checked prior to statistical analyses. Baseline demographic and clinical characteristics of the participants according to groups will be analyzed using descriptive statistics, and inferential statistics will be employed to compare the effects of the dietary interventions on the different outcome measures. Continuous variables following a normal distribution will be expressed as mean ± standard deviation and asymmetrically distributed continuous variables will be expressed as median and interquartile range. Categorical variables will be expressed as absolute and relative frequencies. Departure of genotype distributions from the Hardy-Weinberg equilibrium in all participants will be assessed using chi-square tests. For between-group comparisons, analysis of variance (ANOVA) and the Kruskal-Wallis H test will be used for variables with normal and asymmetric distribution, respectively. The chi-square or Fisher’s exact tests will be used to evaluate categorical variables. All relevant endpoints (new cardiovascular events and death) will be registered as absolute number and percentages according to groups. The generalized estimating equations (GEE) model under the missing-at-random assumption will be used for comparison between outcome variables during the study period. Delta from all outcome data will be obtained from the difference between the measure at 12 weeks and baseline, and will be compared according to groups using analysis of covariance (ANCOVA), adjusted for potential confounders (for instance, differences in baseline data). The SNPs will be included in all ANCOVA models as interaction variables. There is no interim analysis planned. The significance level will be set at 5 %, and all data will be analyzed in SPSS 18.0 (SPSS Inc., Chicago, IL, USA) according to intention-to-treat.

## Discussion

As previously described, the PREDIMED study showed that a MeDiet supplemented with mixed nuts (walnuts, almonds and hazelnuts) or olive oil reduces the incidence of major cardiovascular events in patients at high cardiovascular risk [5]. The PREDIMED study was conducted in Spain; in our study, instead of a classical MeDiet, participants receive a meal plan according to their daily caloric needs (maintenance or reduction of body weight), based on Brazilian guidelines [21–24], and all supplements used in our protocol (olive oil and pecan nuts) are supplied by local producers from southern Brazil. Besides, the amount of daily olive oil prescribed (30 ml/day) to our participants is lower than PREDIMED study, and it was adapted to the dietary habits of Brazilian individuals, who are not used to ingesting very large quantities of olive oil.

It is known that there may be some variations in the chemical composition of olive oil and nuts according to the geographical area where they are grown. Luna et al. [29] evaluated 39 samples of olive oil from eight producing countries; all olive trees were grown under the same conditions and the fruits were harvested at the same stage of maturation. Although the oil was extracted through the same process, these authors identified about 64 different volatile compounds, showing that the geographical area of the olive tree cultivation may be responsible for specific characteristics of olive oil, which could reflect on its beneficial effects [29, 30].

In our study, we are using a Spanish variety of olive oil (*Arbequina*), one that is also cultivated and produced in southern Brazil. A recent study showed that Brazilian olive oils have a high content of phenolic compounds, levels that are comparable to those in olive oils produced in other countries [31]. Noteworthy is the fact that beyond olive oil, the chemical composition of unsaturated fatty acids from pecan nuts is also influenced by its place of cultivation [32].

Although American, European and Asian clinical trials have shown consistent interactions between genetic polymorphisms, diets and the modulation of biochemical cardiovascular markers, there are few reports from Brazilian individuals. Barcelos et al. [33], for instance, showed that the -278A>C (rs3808607) polymorphism in the *CYP7A1* gene can modify serum triglyceride concentrations in response to a reduced fat diet in a dyslipidemic male population; in other study, the consumption of 1 unit of Brazil nuts a day increased the selenium status and the glutathione peroxidase-1 (GPx1) activity in obese women, regardless of the *GPx1* Pro198Leu polymorphism [34]. The Brazilian population is considered one of the most ethnically mixed in the world [35], and this must be considered before all interpretations regarding genetic studies. In populations in which the substructure created by inter-ethnic cross further increases the fluidity of racial and/or ethnic labels, extrapolation of nutrigenetic data from well-defined ethnic groups should be made with caution. To impact positively on global health, nutrigenomics must broaden its scope of investigation with respect to both target and population diversity, to avoid the risk of contributing to the creation of a genomics that is divided between regions and nations.

The randomized controlled GENUTRI trial is designed to explore three different dietary approaches for cardiometabolic and inflammatory profile improvement in patients with established CAD. The dietary approaches suggested by the Brazilian guidelines for individuals at very high cardiovascular risk are largely recommended in our country, and consist of 50–60 % of dairy energy from carbohydrates, 10–15 % from proteins, 25–35 % from total fats, <7 % from saturated fatty acids (SFA), <20 % from MUFA, <10 % from PUFA, <1 % from trans fats, <200 mg/day from dietary cholesterol, 20–30 g/day from fiber and <2400 mg/day of sodium [21–24]. In Brazil, the effects of a modified MeDiet were evaluated by only one small study with 40 CAD patients, with mixed and inconsistent results [36]. The GENUTRI study is the first adequately powered clinical trial focusing on nuts or olive oil supplementation in a southern Brazilian population. Since it may be difficult for Brazilian individuals to adhere to and maintain a traditional MeDiet pattern, the study findings will have important implications not only for our population but also for the feasibility of other potential applications for the long-term prescription of cardioprotective foods in individuals with CAD. Additionally, we will try to demonstrate that genetic polymorphisms linked to cardiovascular disease may modulate the effects of different diets on biochemical and inflammatory markers.

A large randomized clinical trial has been recently proposed in patients with CAD based on novel dietary interventions with the potential for low cost and high feasibility for use in Brazil [37]. However, this protocol does not consider genetic differences regarding the interbred Brazilian population. The GENUTRI trial aims also to stimulate the consumption of local foods since the olive oil and pecan nuts provided in the study are cultivated and produced in our country by farmers on a small scale. If effective, it may contribute to economic growth in southern Brazil by encouraging the consumption of these foods. Besides, our results may generate hypotheses for larger randomized clinical trials regarding nutrigenetic approaches in different Brazilian regions.

In conclusion, although many studies have demonstrated the beneficial effects of the MeDiet and/or dietary intake of olive oil and nuts on cardiometabolic and inflammatory markers, clinical trials evaluating these foods in Brazilian/non-Mediterranean populations with CAD are scarce. Besides, the assessment of specific genetic polymorphisms may help to understand the individual different responses to specific dietary intervention.

### Trial status

The trial is ongoing. One hundred and twenty patients have completed the study protocol and additional patients are being recruited. The results of the study will be communicated to all participants.

## Abbreviations

24-HDRs: Dietary recalls
AHA: American Heart Association
CAD: Coronary artery disease
*CD36*: CD36 molecule (thrombospondin receptor) gene
GCP: Good Clinical Practice
HDL-c: High-density lipoprotein cholesterol
hs-CRP: High-sensitivity C-reactive protein
IL-10: Interleukin-10
IL-6: Interleukin-6
IPAQ: International Physical Activity Questionnaire
LDL-c: Low-density lipoprotein cholesterol
MeDiet: Mediterranean Diet
PREDIMED: PREvención con DIeta MEDiterránea
SNP: Single-nucleotide polymorphism
*STAT3*: Signal transducer and activator of transcription 3 (acute-phase response factor) gene
TNF-α: Tumor necrosis factor alpha
WHO: World Health Organization

## References

[1] Murray CJ, Vos T, Lozano R, Naghavi M, Flaxman AD, Michaud C (2012). Disability-adjusted life years (DALYs) for 291 diseases and injuries in 21 regions, 1990–2010: a systematic analysis for the Global Burden of Disease Study 2010. Lancet.

[2] Wong ND (2014). Epidemiological studies of CHD and the evolution of preventive cardiology. Nat Rev Cardiol.

[3] World Health Organization (2011). World Heart Federation and World Stroke Organization. Global Atlas on Cardiovascular Disease Prevention and Control.

[4] Jankovic N, Geelen A, Streppel MT, de Groot LC, Orfanos P, van den Hooven EH (2014). Adherence to a healthy diet according to the World Health Organization guidelines and all-cause mortality in elderly adults from Europe and the United States. Am J Epidemiol.

[5] Estruch R, Ros E, Salas-Salvadó J, Covas MI, Corella D, Arós F (2013). Primary prevention of cardiovascular disease with a Mediterranean diet. N Engl J Med.

[6] Sofi F, Abbate R, Gensini GF, Casini A (2010). Accruing evidence on benefits of adherence to the Mediterranean diet on health: an updated systematic review and meta-analysis. Am J Clin Nutr.

[7] Nordmann AJ, Suter-Zimmermann K, Bucher HC, Shai I, Tuttle KR, Estruch R (2011). Meta-analysis comparing Mediterranean to low-fat diets for modification of cardiovascular risk factors. Am J Med.

[8] Trichopoulou A, Kouris-Blazos A, Wahlqvist ML, Gnardellis C, Lagiou P, Polychronopoulos E (1995). Diet and overall survival in elderly people. BMJ.

[9] Casas R, Sacanella E, Urpí-Sardà M, Chiva-Blanch G, Ros E, Martínez-González MA (2014). The effects of the Mediterranean diet on biomarkers of vascular wall inflammation and plaque vulnerability in subjects with high risk for cardiovascular disease. A randomized trial. PLoS One.

[10] Luo C, Zhang Y, Ding Y, Shan Z, Chen S, Yu M (2014). Nut consumption and risk of type 2 diabetes, cardiovascular disease, and all-cause mortality: a systematic review and meta-analysis. Am J Clin Nutr.

[11] Schwingshackl L, Hoffmann G (2014). Monounsaturated fatty acids, olive oil and health status: a systematic review and meta-analysis of cohort studies. Lipids Health Dis.

[12] Degirolamo C, Rudel LL (2010). Dietary monounsaturated fatty acids appear not to provide cardioprotection. Curr Atheroscler Rep.

[13] Albert CM, Gaziano JM, Willett WC, Manson JE (2002). Nut consumption and decreased risk of sudden cardiac death in the Physicians’ Health Study. Arch Intern Med.

[14] Franchini M, Peyvandi F, Mannucci PM (2008). The genetic basis of coronary artery disease: from candidate genes to whole genome analysis. Trends Cardiovasc Med.

[15] Knowles JW, Wang H, Itakura H, Southwick A, Myers RM, Iribarren C (2007). Association of polymorphisms in platelet and hemostasis system genes with acute myocardial infarction. Am Heart J.

[16] Aaronson DS, Horvath CM (2002). A road map for those who don’t know JAK-STAT. Science.

[17] Keller KL, Liang LC, Sakimura J, May D, van Belle C, Breen C (2012). Common variants in the CD36 gene are associated with oral fat perception, fat preferences, and obesity in African Americans. Obesity (Silver Spring).

[18] Phillips CM, Goumidi L, Bertrais S, Field MR, Peloso GM, Shen J (2009). Dietary saturated fat modulates the association between STAT3 polymorphisms and abdominal obesity in adults. J Nutr.

[19] Cannon CP, Brindis RG, Chaitman BR, Cohen DJ, Cross JT, Drozda JP (2013). 2013 ACCF/AHA key data elements and definitions for measuring the clinical management and outcomes of patients with acute coronary syndromes and coronary artery disease. Circulation.

[20] WHO. Guidelines for Good Clinical Practice (GCP) for Trials on Pharmaceutical Products. Geneva; 1995. p. 97–137. (Technical Report Series no. 850)

[21] Sociedade Brasileira de Hipertensão, Sociedade Brasileira de Cardiologia, Sociedade Brasileira de Endocrinologia e Metabologia, Sociedade Brasileira de Diabetes, Sociedade Brasileira de Estudos da Obesidade (2005). I Brazilian guidelines on diagnosis and treatment of metabolic syndrome. Arq Bras Cardiol.

[22] Sociedade Brasileira de Cardiologia (2013). V Diretriz Brasileira Sobre Dislipidemias e Prevenção da Aterosclerose Departamento de Aterosclerose da Sociedade Brasileira de Cardiologia. Arq Bras Cardiol.

[23] Sociedade Brasileira de Cardiologia, Sociedade Brasileira de Hipertensão, Sociedade Brasileira de Nefrologia (2010). VI Brazilian guidelines on hypertension. Arq Bras Cardiol.

[24] Sociedade Brasileira de Diabetes (2009). Diretrizes da Sociedade Brasileira de Diabetes.

[25] Kris-Etherton PM, Innis S, American Dietetic Association, Dietitians of Canada (2007). Position of the American Dietetic Association and Dietitians of Canada: dietary fatty acids. J Am Diet Assoc.

[26] U.S. Department of Agriculture and U.S. Department of Health and Human Services (2010). Dietary Guidelines for Americans, 2010.

[27] Avanutri Revolution® Software. Available at: http://www.avanutri.com.br/softwares. Accessed July 2016.

[28] Craig CL, Marshall AL, Sjöström M, Bauman AE, Booth ML, Ainsworth BE (2003). International physical activity questionnaire: 12-country reliability and validity. Med Sci Sports Exerc.

[29] Luna G, Morales MT, Aparicio R (2006). Characterisation of 39 varietal virgin olive oils by their volatile compositions. Food Chem.

[30] Bakhouche A, Lozano-Sánchez J, Beltrán-Debón R, Joven J, Segura-Carretero A, Fernández-Gutiérrez A (2013). Phenolic characterization and geographical classification of commercial Arbequina extra-virgin olive oils produced in southern Catalonia. Food Res Int.

[31] Ballus CA, Quirantes-Piné R, Bakhouche A, da Silva LF, de Oliveira AF, Coutinho EF (2015). Profile of phenolic compounds of Brazilian virgin olive oils by rapid resolution liquid chromatography coupled to electrospray ionisation time-of-flight mass spectrometry (RRLC-ESI-TOF-MS). Food Chem.

[32] Venkatachalam M, Kshirsagar HH, Seeram NP, Heber D, Thompson TE, Roux KH (2007). Biochemical composition and immunological comparison of select pecan [Carya illinoinensis (Wangenh.) K. Koch] cultivars. J Agric Food Chem.

[33] Barcelos ALV, Chies R, Almeida SEM, Fiegenbaum M, Schweigert ID, Chula FGL (2009). Association of CYP7A1 -278A>C polymorphism and the response of plasma triglyceride after dietary intervention in dyslipidemic patients. Braz J Med Biol Res.

[34] Cominetti C, de Bortoli MC, Purgatto E, Ong TP, Moreno FS, Garrido AB (2011). Associations between glutathione peroxidase-1 Pro198Leu polymorphism, selenium status, and DNA damage levels in obese women after consumption of Brazil nuts. Nutrition.

[35] Suarez-Kurtz G (2005). Pharmacogenomics in admixed populations. Trends Pharmacol Sci.

[36] Thomazella MC, Góes MF, Andrade CR, Debbas V, Barbeiro DF, Correia RL (2011). Effects of high adherence to Mediterranean or low-fat diets in medicated secondary prevention patients. Am J Cardiol.

[37] Weber B, Bersch-Ferreira AC, Torreglosa CR, Ross-Fernandes MB, Silva JC, Galante AP (2016). The Brazilian Cardioprotective Nutritional Program to reduce events and risk factors in secondary prevention for cardiovascular disease: study protocol (The BALANCE Program Trial). Am Heart J.

